# Supplementary material for: P2Y2 and P2X4 Receptors Mediate Ca2+ Mobilization in DH82 Canine Macrophage Cells
Source: Int J Mol Sci. 2020 Nov 13;21(22):8572. doi: 10.3390/ijms21228572 (PMC7696671; doi:10.3390/ijms21228572)
Supplement: Supplementary file 1 [file ijms-21-08572-s001.zip › Sophocleous_ et_al_Supplementary.docx]

Supplementary data

P2Y_2_ and P2X4 receptors mediate Ca^2+^ mobilization in DH82 canine macrophage cells

Reece Andrew Sophocleous ^1,2^, Nicole Ashleigh Miles ^1,2^, Lezanne Ooi ^1,2^ and Ronald Sluyter ^1,2,*^

^1^ Illawarra Health and Medical Research Institute, Wollongong, NSW, Australia; rs256@uowmail.edu.au (R.A.S.); nam993@uowmail.edu.au (N.A.M.); lezanne@uow.edu.au (L.O.)

^2^ Molecular Horizons and School of Chemistry and Molecular Bioscience, University of Wollongong, Wollongong, NSW, Australia

***** Correspondence: rsluyter@uow.edu.au; Tel.: +612-4221-5508

**Table S1.** Primers for RT-PCR amplification of DH82 cell cDNA

| **Gene target/ exon** | **Forward primer (5’-3’)** | **Reverse primer (5’-3’)** | **Annealing conditions** | **Product size (bp)** |
| --- | --- | --- | --- | --- |
| *P2RX1* | TGGTGCAAGAGTCAGGACAG | ATTGGTCCCATTTTCCACAA | 50°C for 30 s | 206 |
| *P2RX2* | CAGTTCTCCAAGGGCAACAT | GTCCAGGTCACAGTCCCAGT | 56°C for 30 s | 195 |
| *P2RX3* | CTTGCACGAGAAGGCTTACC | ATCCTTGCATCTGGTTTTCG | 51.5°C for 30 s | 188 |
| *P2RX4* | CAACCTGGACAGAACCTCCTC | CGCACGCTCATCAAGGCCTA | 58°C for 30 s | 159 |
| *P2RX5* | TCCGCACGCTGATGAAAGCCTA | AAGAAAGCCCCAGCACCCATGA | 60°C for 30 s | 133 |
| *P2RX6* | ATTCAACGGTACCCACAGGA | AGTAAGTGGCATCCCAGGTG | 54.5°C for 30 s | 185 |
| *P2RX7* | TGCCTCCCATCCCAGCTCCC | GGCGCTTTGGCTCCCAGGAC | 60.8°C for 30 s | 240 |
| *P2RY1* | TCGTGCTCATCCTGGGCTGCTA | TGTAGGACACGGCGAAGACCGT | 61°C for 30 s | 132 |
| *P2RY2* | TTTGTCACCACCAGCGTGCG | AGCGCGTAGCACACCAGGAT | 59°C for 30 s | 149 |
| *P2RY4* | TCAGCTCGGCAATCATGGGGTT | TGCGGTTGATGTGGAAAGGCAC | 58°C for 30 s | 195 |
| *P2RY6* | ACAGGCATCCAGCGTAATCGCA | AAAGGGCAGCAGAAAGCCGATGA | 59°C for 30 s | 114 |
| *P2RY11* | ACAAGGCATTGAGACCAGCAGCAG | TGGTCCCCGAACAGGTCTTTGTGT | 60°C for 30 s | 138 |
| *P2RY12* | AGTGATGCCAAGCTGGGAACAGGA | AGGTTGTTGGGGCTGGACGTCTTA | 60°C for 30 s | 167 |
| *P2RY13* | TCACCGGCATCCTGTTGAACACT | ACACGAAGGCTCTGAGTTGCCA | 58°C for 30 s | 180 |
| *P2RY14* | AGGGTCTCCGCTGTGCTCTTCTAT | AGCATGAGCACCCACACCATCA | 59°C for 30 s | 167 |
| *GAPDH* | GGAGAAAGCTGCCAAATATG | ACCAGGAAATGAGCTTGACA | 49°C for 30 s | 200 |


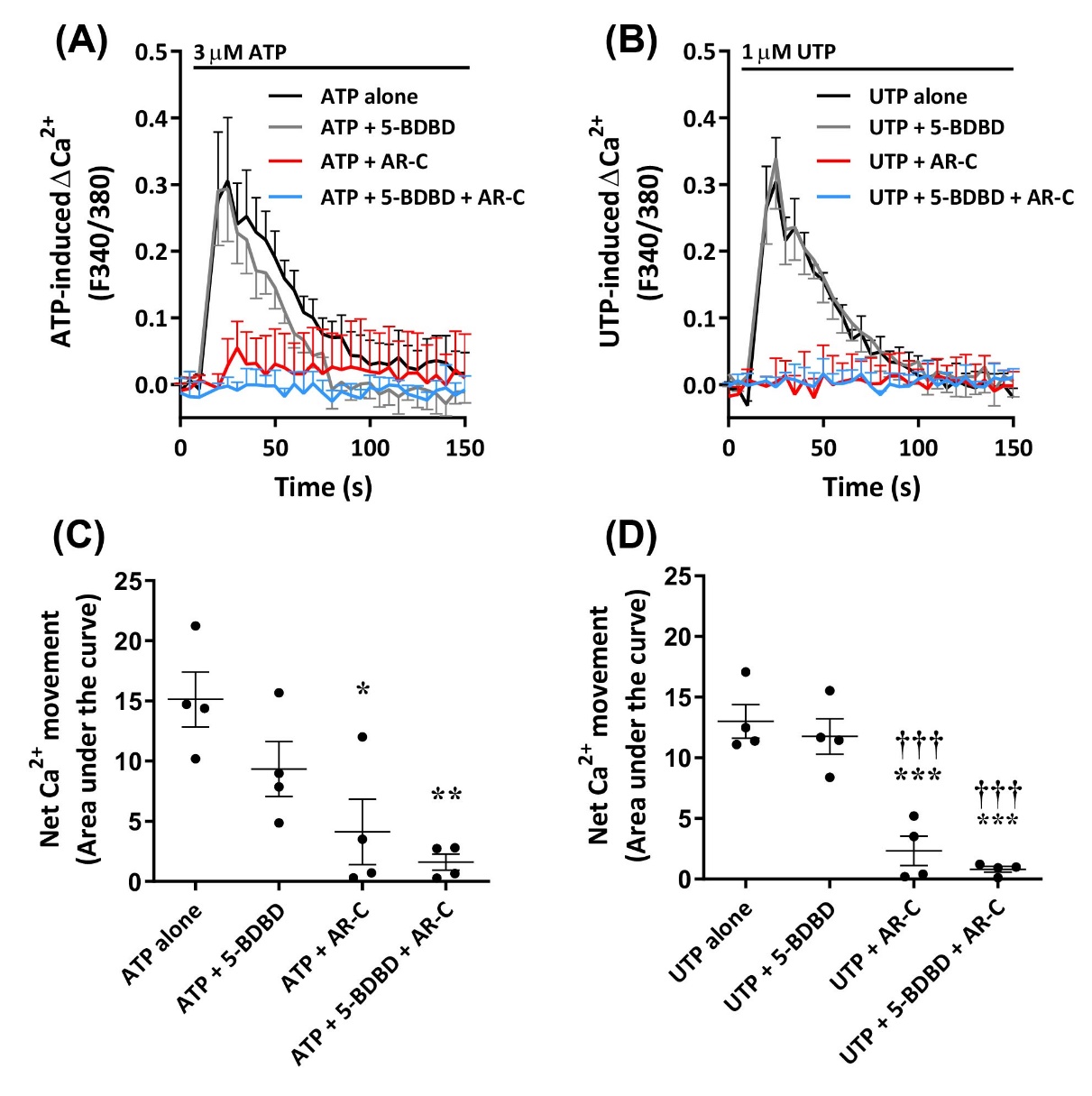


**Figure S1.** Combined inhibition of ATP- and UTP-induced Ca^2+^ responses in DH82 cells preincubated with 5-BDBD and AR-C118925. (A-D) DH82 cells in ECS were loaded with Fura-2 and preincubated in the absence or presence of 30 µM 5-BDBD (0.3% DMSO) and/or 10 µM AR-C118925 (AR-C; 0.03% DMSO) for 30 minutes. Cells were then exposed to (A) 3 µM ATP or (B) 1 µM UTP (respective ~EC_80_ values) and Fura-2 fluorescence was recorded. (C) ATP-induced and (D) UTP-induced net Ca^2+^ movement were calculated as area under the curve from (A) and (B), respectively. (A-D) Data shown are mean ± SEM from four independent experiments. ^*^*P* < 0.05, ^**^*P* < 0.01 and ^***^*P* < 0.001 compared to respective nucleotide alone; ^††^*P* < 0.01 and ^†††^*P* < 0.001 compared to respective nucleotide + 5-BDBD analysed using a one-way ANOVA with Bonferroni *post hoc* test.


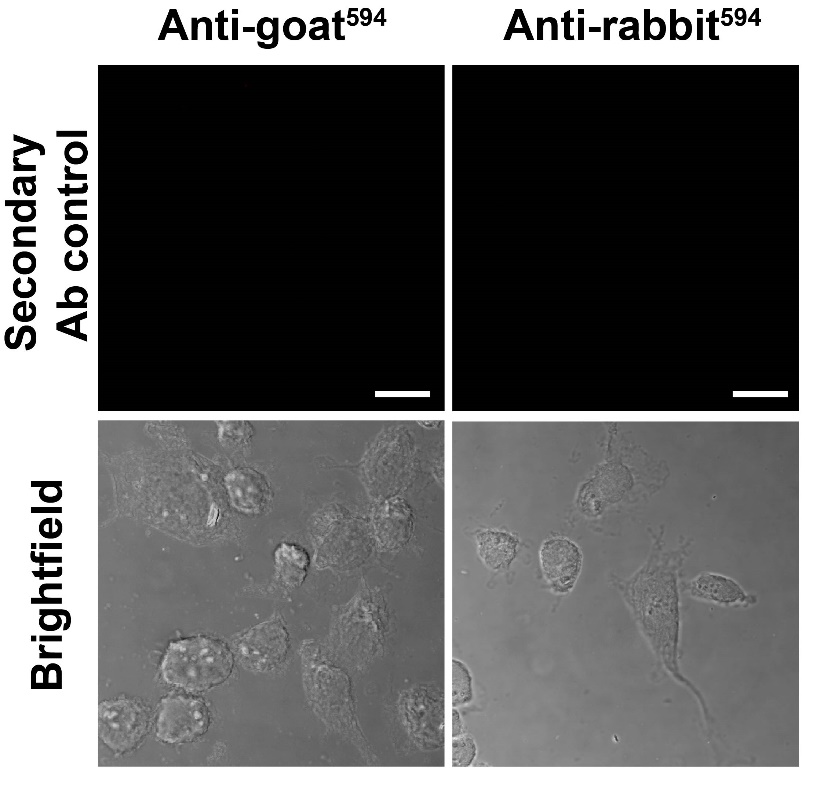


**Figure S2. Secondary antibody controls for anti-P2X4 receptor and anti-P2Y2 receptor antibodies in DH82 cells.** DH82 cells were fixed, permeabilized and stained with anti-goat^594^ or anti-rabbit^594^ secondary antibodies. Cells were imaged by confocal microscopy. Images are representative results of three separate experiments. Scale bar = 20 µM.

| 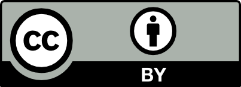 | © 2020 by the authors. Submitted for possible open access publication under the terms and conditions of the Creative Commons Attribution (CC BY) license (http://creativecommons.org/licenses/by/4.0/). |
| --- | --- |
